# SpasticSim: a synthetic data generation method for upper limb spasticity modelling in neurorehabilitation

**DOI:** 10.1038/s41598-024-51993-w

**Published:** 2024-01-18

**Authors:** Rubén de-la-Torre, Edwin Daniel Oña, Juan G. Victores, Alberto Jardón

**Affiliations:** Department of Systems Engineering and Automation, Universidad Carlos III de Madrid, Avda. de la Universidad 30, Leganés, 28911 Madrid, Spain

**Keywords:** Engineering, Neurological disorders, Experimental models of disease, Preclinical research, Diagnosis, Movement disorders

## Abstract

In neurorehabilitation, assessment of functional problems is essential to define optimal rehabilitation treatments. Usually, this assessment process requires distinguishing between impaired and non-impaired behavior of limbs. One of the common muscle motor disorders affecting limbs is spasticity, which is complicated to quantify objectively due to the complex nature of motor control. Thus, the lack of heterogeneous samples of patients constituting an acceptable amount of data is an obstacle which is relevant to understanding the behavior of spasticity and, consequently, quantifying it. In this article, we use the 3D creation suite Blender combined with the MBLab add-on to generate synthetic samples of human body models, aiming to be as sufficiently representative as possible to real human samples. Exporting these samples to OpenSim and performing four specific upper limb movements, we analyze the muscle behavior by simulating the six degrees of spasticity contemplated by the Modified Ashworth Scale (MAS). The complete dataset of patients and movements is open-source and available for future research. This approach advocates the potential to generate synthetic data for testing and validating musculoskeletal models.

## Introduction

Spasticity is a motor disorder that causes stiffness or tightness of muscles, and can alter the normal functioning of muscles and joints. The nature of this condition is complex, and a variety of definitions of spasticity have been suggested. In 1980, Lance^1^ defined spasticity as a motor disorder characterized by an increase in tonic stretch reflexes with exaggerated tendon jerks, resulting from hyper-excitability of the stretch reflex. However, this definition does not fully encompass the complexities of motor control. In 2005, the Support Program for Assembly of a Database for Spasticity Measurement (SPASM) project defined spasticity as “disordered sensory-motor control resulting from an upper motor neuron lesion, presenting as intermittent or sustained involuntary activation of muscles”^2^. This definition highlights the complex character of spasticity due to the heterogeneity of symptoms and the nature of motor control.

While it is difficult to find a holistic definition of spasticity, it is also difficult to objectively measure the affectation level of this phenomenon. For this purpose, some scales were created to evaluate the level of the disorder, such as the modified Ashworth scale (MAS), the Tardieu scale, and the Spasm severity scale, among others^3^. Currently, these scales are still the gold standard in clinical practice; however, they are based on the perception of the clinician that evaluates the patient’s spasticity through manual mobilization of the limb, experience, and training over the years^4,5^. Therefore, the development of instrumented or automated methods for spasticity assessment in neurorehabilitation has been increasing in the past 20 years^6^.

One of the major challenges in developing technical aids for neurorehabilitation is dealing with the heterogeneity of patients’ characteristics. Validation studies include randomized clinical trials (RCT) with sample sizes as large as possible in order to obtain significant insights. However, patient recruitment is sometimes difficult, and, in consequence, most of the evidence comes from small sample size studies^7,8^, often leading to non-generalizable conclusions. Thus, sample size is one of the most relevant factors for the quality of the investigation^9,10^, and determines the scope of the study. Appropriate sample sizes directly impact the quality of outcomes^11,12^ and will allow the development of more comprehensive analytical models^13,14^. Nevertheless, convenient data is not accessible for public analysis and hinders future valuable work.

This study seeks to obtain a heterogeneous variety of data which will help to properly model spasticity, and propose a new method to generate synthetic data for healthcare applications. Exploring feasible alternatives, a dataset for this research was developed using a 3D modeling software with an add-on to simulate different body configurations. By employing this method, we can produce as many patients as the study demands, modifying the physical characteristics to produce the desired batch. A search of the literature revealed few studies which attempted to investigate synthetic data in this area of the healthcare field, but there have been several advances that confirm an increasing tendency in the field as a whole^15,16^.

The method proposed in this paper aims to contribute to this area and the filed of healthcare in general because it acknowledges the profitable use of synthetic data for the creation of human body models, and allows the reproduction not only of spasticity in the upper limb (UL) but it also for different musculoskeletal behaviors in other body joints. For this purpose, the complete dataset generated in this study is available for other researchers.

The remainder of this paper is as follows. “Background” presents a brief review of related work on the topic of this manuscript. “Methodology” describes the methodology employed in this study. “Synthetic dataset generation” details the procedure to create the synthetic data for spasticity modelling. “Proposed framework for spasticity modelling” presents an application case of the proposed dataset in the modelling of spasticity, evaluating preliminary the feasibility of this method. Finally, the obtained results are discussed in “Discussion”, and the conclusions are presented in “Conclusions”.

## Background

Synthetic data or data that is artificially generated has been used successfully in a variety of applications. For example, systems based on statistical testing involve generating synthetic test data. Such data must possess the same statistical characteristics as the actual data that the system will process during operation^17^. In this line, the study conducted by Soltana et al.^17^ presents and evaluates a method to generate a synthetic population of citizens’ records for testing a public administration IT system. In the case of Internet of Things (IoT) applications, data can be limited by issues about the release of privately owned information. In this context, the use of synthetic data could reduce such a limitations, being an alternative that exhibits the complex characteristics of original data without compromising proprietary data and personal privacy^18^. Similarly, financial services generate a large volume of complex and varied data; however, the available datasets are scarce due to regulatory issues or business needs. Thus, the financial domain demands methods for effective synthetic data generation^19^.

Another example is robotic and computer vision problems, where most of the current datasets and environments lack realism, interactions, and details from the real world^20^. A manner to reduce this drawback is the use of hyperrealistic virtual environments, e.g. with indoor scenes to be explored by robot agents^21^. This type of studies highlights the potential of game engines to generate synthetic data, which facilitates training data-driven methods.

In the healthcare domain, synthetic data is gaining more attention in recent years because of its potential in making timely healthcare data more accessible for analysis and technology development^22^. An example application is the training of deep learning models for action recognition of elders’ daily activities, where large-scale activity datasets are needed. The use of synthetic data enables the generation of large-scale realistic motions, with various adjustable features to train human action recognition models^23^.

Another example is presented in the Loecher et al. study^24^, where large amounts of training data were created using natural images to automate cardiac MRI tag tracking with a convolutional neural network (CNN). Moreover, the Dahmen et al. study introduce SynSys, a machine learning-based synthetic data generation method to increase complexity and realism of behaviour-based sensor data for healthcare applications^25^. The results suggests that this technique can improve activity recognition accuracy in comparison with small size real data. Other synthetic data generation strategies in the healthcare domain are summarised in the Murtaza et al. review^26^.

On account of the above, it seems clear the potential of clinical data synthesis to generate realistic data for healthcare research, where real-world data is difficult to obtain or unnecessary. However, it seems also clear the concerns about synthetic data validity in healthcare applications. The Chen et al. study fills this gap by calculating clinical quality measures using synthetic data^27^. Results show that synthetic patient generators are quite reliable in modeling demographics and other features in healthcare settings inspiring with its differences a new method to simulate spasticity.

Overall, synthetic data can be classified into three broad categories: fully synthetic (it contains no original data)^28^, partially synthetic (it replaces the values of a few sensitive attributes with synthetic values), and hybrid (it is created using both real and made-up information)^29^. Nevertheless, the original real data remains the preferred choice. However, most datasets containing health information are not readily available for use because they contain confidential information about individuals. Hence, synthetic data holds possibilities in bridging data access gaps in research and evidence-based policymaking^22^. Synthetic data addresses three challenges in making healthcare data accessible: protection of individual’s privacy in datasets, accessibility to healthcare research data, and reducing the lack of realistic data for software development and testing^22^.

## Methodology

**Figure 1:**
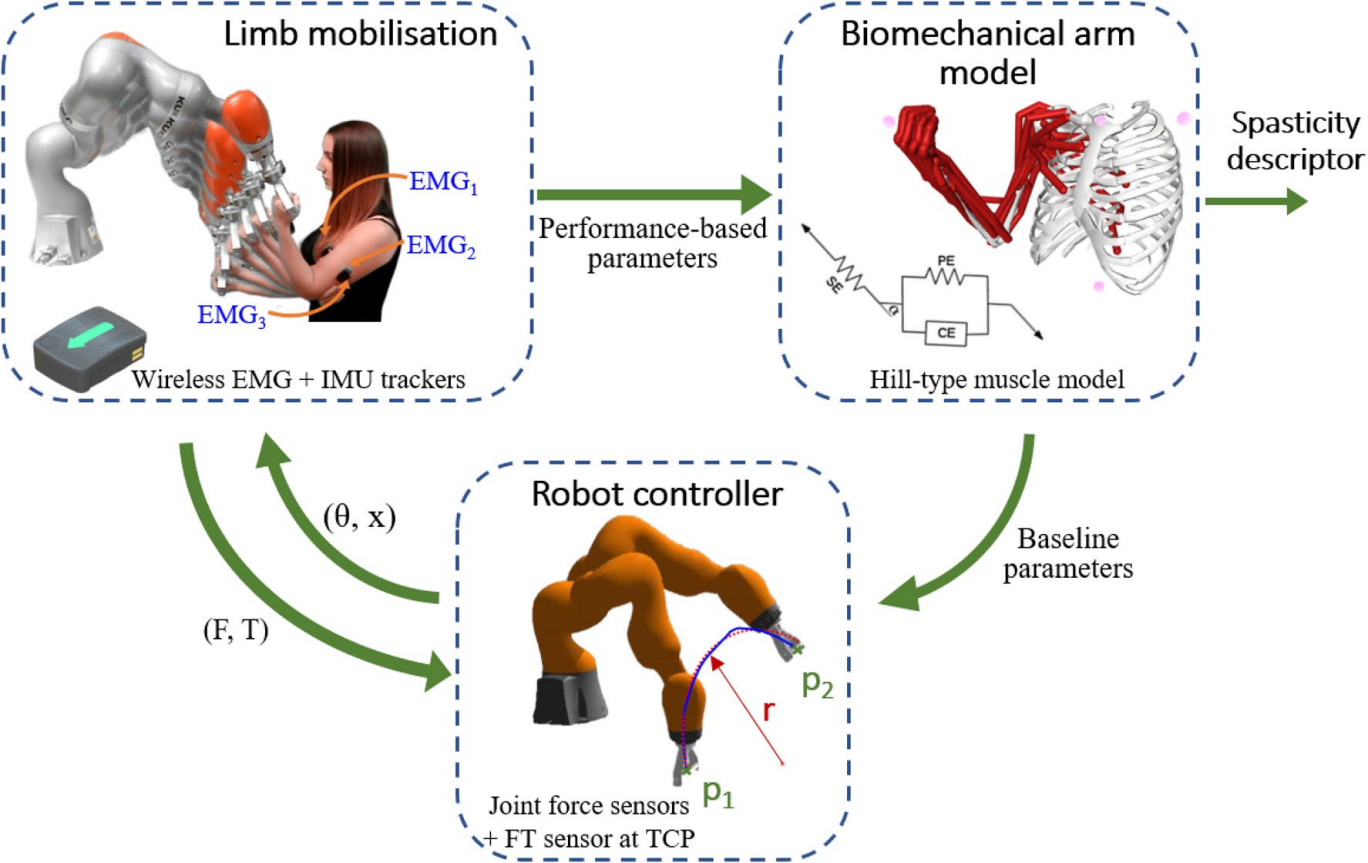
Robot-aided spasticity assessment.

The scope of this work is the development of a robot-aided system for autonomous evaluation of spasticity level in the upper extremities^30^. Based on the clinical practice, the proposed system promote arm mobilisations assisted by a robotic arm, and gather much more information about the user performance during the interaction. This raw data feeds a biomechanical model of the arm in order to detect the behaviour of a spastic muscle. Figure 1 describes the process for the robot-aided spasticity assessment. The proposed system seems to offer various advantages regarding traditional manual procedures. However, there are still issues to overcome, as the development of a classifier to differentiate between movements with spasticity, which requires a large amount of data for properly training.

On account of the above, this study aims to (1) create a dataset with synthetic data of human individuals performing different UL movements, and (2) propose a framework for modelling UL impairments, such as spasticity, based on such a dataset.

On the one hand, the dataset development comprises two main steps:

- Generation of synthetic data of the human body with different demographic characteristics using a 3D modelling software. For this study, the open source 3D creation suite Blender^31^ is employed because it is a powerful tool for 3D pipeline-modeling, rigging, animation, simulation, rendering, compositing and motion tracking, even video editing and game creation. Not only Blender was selected for its reliability and validity but it also because of the amount of open source information. Blender does not requires a specific software training and the public work allows self-learning to develop the dataset.
- Motion addition to the 3D human models to include dynamics to the target limb. For that purpose, the MB-Lab tool is used^32^. MB-Lab is a Blender add-on that creates humanoid characters, allowing to customise several features such as phenotypes, facial expressions, and body movement animations using inverse kinematics.

As a result, a synthetic dataset including plenty of human features and upper limb motion is available. However, the generic procedure proposed in this paper (3D modelling plus motion addition) allows to customize the dataset according to another target limb or population.

On the other hand, the interest in using a synthetic dataset is the possible application in motor impairments diagnoses. The lack of real patient data to analyse the motor control behaviour is a relevant issue to elaborate health strategies to understand, measure, and diagnose the motor control impairments. Therefore, using state-of-the-art simulation software for modeling and analysis of movement could contribute to advances in research despite not having real patient data but realistic samples. As a study case, this paper proposes a framework to develop a spasticity model using the musculoskeletal simulation software OpenSim. This spasticity model may help to develop a classifier trained with synthetic data but reliable enough to estimate the spasticity level in human patients.

## Synthetic dataset generation

**Figure 2:**
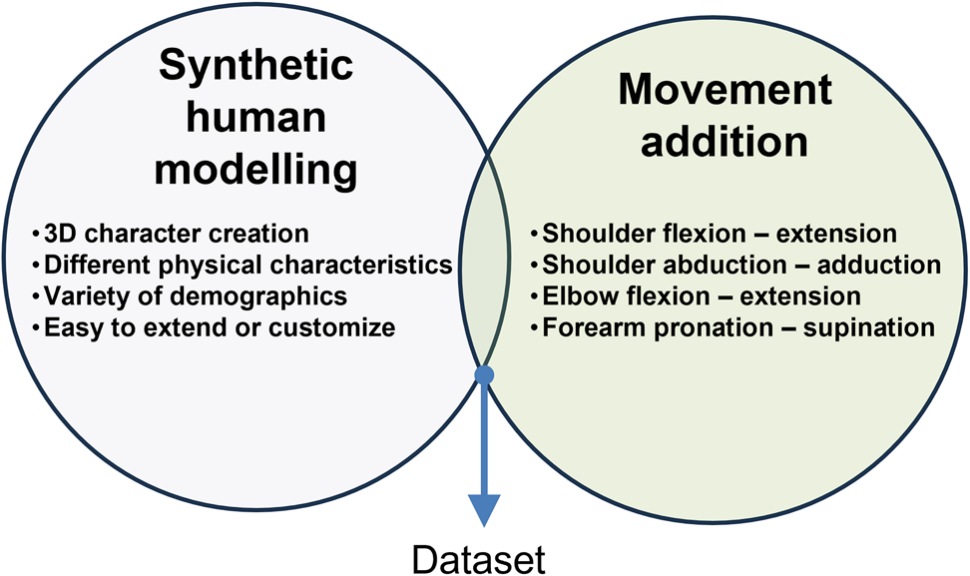
Schema of dataset creation.

The process to create the synthetic dataset is divided in two steps: the customisation of character, and the addition of motion to the target limb. The Blender software version 2.92 and MB-Lab add-on version 1.7.8 are used in both steps. A scheme of the process to generate the dataset is illustrated in Fig. 2.

### Synthetic human body models

Human model data were generated using Blender, a 3D modelling software mostly used for computer animation. This tool allows the creation of human patients with different physical characteristics such as height, mass, weight, bone length or muscular tone. Codd et al.^33^ assesses the efficacy of Blender as a teaching tool of human forearm musculoskeletal anatomy and Hamid et al.^34^ assesses the computational speed of solvers in a commercial, low end laptop. In addition, we were also employing a special complement designed for Blender named MB-Lab developed by Manuel Bastioni^32^. Both software are open-source and not difficult to manage due to the multiple tutorials and the extended use in the field of computer-generated imagery.

On this basis, we have chosen Blender software to create human synthetic samples aiming to be representative enough as human ones. Different authors have studied the validity of synthetic samples in a variety of applications. Zhao et al.^35^ confirmed that “Virtual Reality (VR) may act as an efficient way to improve the learners level of anatomy knowledge” and Timohir et al.^36^ established that “a way was sought to improve the possibilities for 3D modeling of anatomical people in accordance with the anthropometric features”. The studies presented thus far provide evidence that synthetic human models can be used as proper samples in our investigation.

The created sample intend to be diverse with respect to gender, age, height, mass, tone and pursue to include the human diversity around the world. For this reason, we created 92 synthetic individuals listed in Table 1. The subjects fulfill our main objective of a heterogeneous dataset and contain the necessary data for an accurate modeling. The key aspects of every synthetic patient are genotype, age, height, mass, tone, upper arm length, forearm length, and hand length.

The genotype category MB-Lab standard library covers the most common human ethnic phenotypes, such as Caucasian, African, Asian, and Latino people. Latino Male and Latino Female have the same height, upper arm length, forearm length and hands length than the Caucasian male and Caucasian female types. For this reason, note that we included only two individuals with different ages just to consider also the Latino type.

Changing the genotype and age of the patient will result in a predefined upper arm length, forearm length, hand length, and height assigned automatically by MB-Lab. These body measures have been preset in the software MB-Lab and for our study, we are taking into consideration just the standard output of the software. However, the values can be modified in Blender, and adjust the lengths or the height due to the needs of the investigation.

Changing the age of any of the 4 predefined phenotypes will also automatically vary the height and body measures of the created sample. Adulthood is usually divided into 3 main periods: early adulthood (approximately aged 20–39), middle adulthood (40–59), and old age (60+). For this reason, 3 different ages as 23, 48 and 68 were chosen to integrate the complete adulthood life.

**Figure 3:**
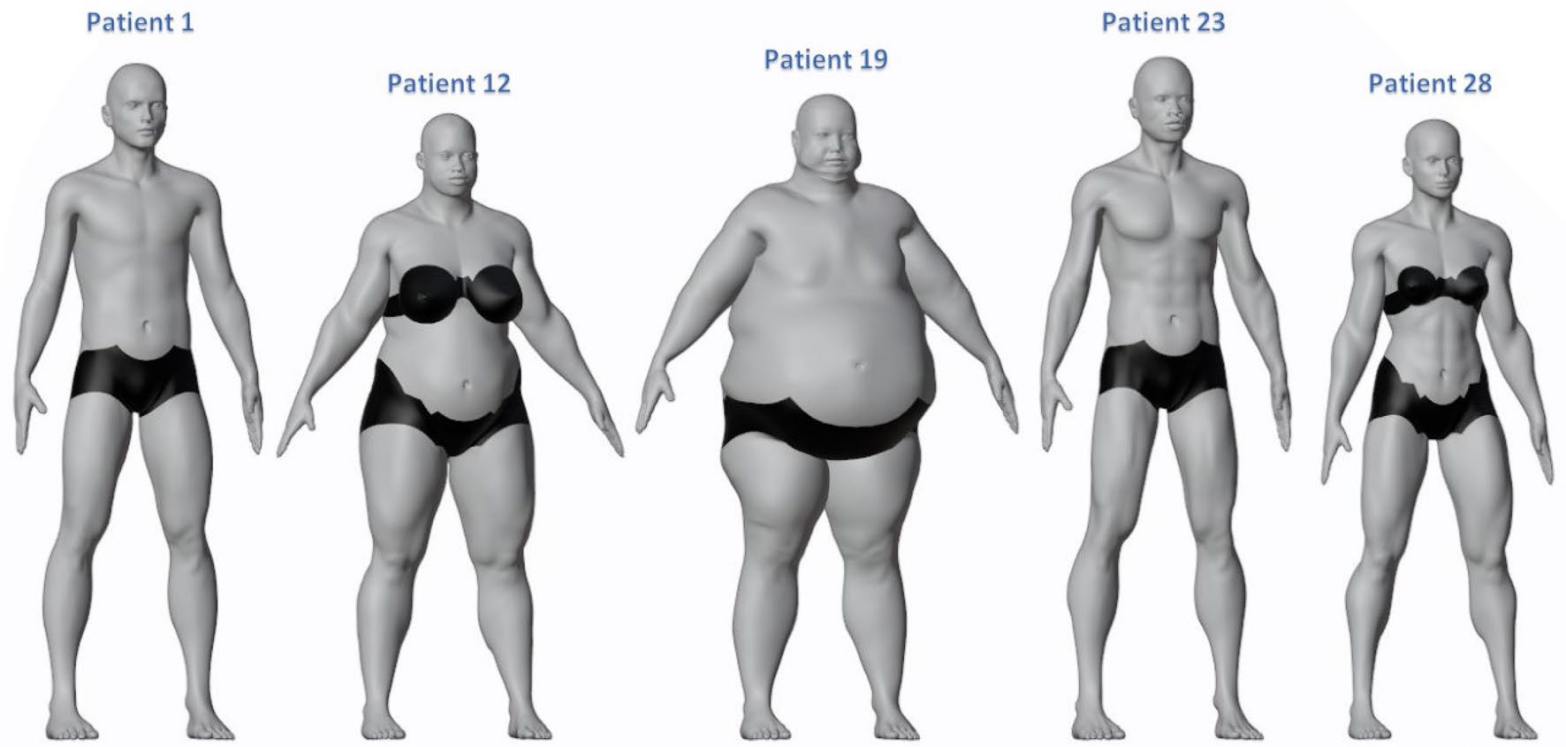
Mass and tone diversity. Patient 1: 50% mass and 50% tone; patient 12: 75% mass and 50% tone; patient 19: 75% mass and 25% tone; patient 23: 25% mass and 75% tone; patient 28: 50% mass and 75% tone.

Additionally, Fig. 3 depicts the percentages of Mass and Tone used for dataset generation. Those percentages were selected attempting to include all types of human bodies and excluding limited percentages under 25% or above 75% because extreme and atypical models, even existing, are not representative of the usual population. Therefore, the beginning of the research started with 50% mass and 50% tone, and a variation of 25% in mass and tone was included it considering those archetypes as representative.

**Table 1:** Summary of synthetic patient demographics to build the dataset.

ID	Type	Age	Height	Mass	Tone	Upperarm	Forearm	Hands
1	Caucasian Male	23	179.6	50	50	28.5	28.62	21.3
2	Caucasian Female	23	164.9	50	50	29.38	24.24	18.35
3	African Male	23	181.4	50	50	28.76	28.96	21.54
4	African Female	23	166.4	50	50	29.49	24.33	18.41
5	Asian Male	23	171.46	50	50	27.15	27.05	20.23
6	Asian Female	23	158.52	50	50	28.25	23.31	17.83
7	Latino Male	33	182.6	50	50	29.04	29.12	21.37
8	Latino Female	33	161.3	50	50	28.78	23.65	17.74
9	Caucasian Male	23	179.6	75	50	28.26	29.32	21.44
10	Caucasian Female	23	164.9	75	50	30.17	24.65	18.39
11	African Male	23	181.4	75	50	28.5	29.73	21.67
12	African Female	23	166.4	75	50	30.28	24.74	18.45
13	Asian Male	23	171.46	75	50	26.98	27.83	20.37
14	Asian Female	23	158.52	75	50	29.04	23.73	17.69
15	Caucasian Male	23	179.6	75	25	28.32	30.03	21.3
16	Caucasian Female	23	164.9	75	25	31.32	23.76	18.36
17	African Male	23	181.4	75	25	28.55	30.37	21.53
18	African Female	23	166.4	75	25	31.42	23.85	18.42
19	Asian Male	23	171.46	75	25	27.05	28.48	20.23
20	Asian Female	23	158.52	75	25	30.18	22.84	17.66
21	Caucasian Male	23	179.6	25	75	28.58	28.53	21.29
22	Caucasian Female	23	164.9	25	75	29.46	24.19	18.34
23	African Male	23	181.4	25	75	28.84	28.87	21.52
24	African Female	23	166.4	25	75	29.57	24.28	18.4
25	Asian Male	23	171.46	25	75	27.23	26.96	20.22
26	Asian Female	23	158.52	25	75	28.33	23.26	17.64
27	Caucasian Male	23	179.6	50	75	28.36	28.73	21.43
28	Caucasian Female	23	164.9	50	75	29.3	24.81	18.38
29	African Male	23	181.4	50	75	28.61	29.07	21.67
30	African Female	23	166.4	50	75	29.4	24.9	18.45
31	Asian Male	23	171.46	50	75	27.03	27.16	20.37
32	Asian Female	23	158.52	50	75	28.16	23.89	17.69
33	Caucasian Male	68	178.1	50	50	28.79	28.79	20.95
34	Caucasian Female	68	163.3	50	50	29.62	24.12	18.17
35	African Male	68	179.9	50	50	29.05	29.14	21.18
36	African Female	68	164.81	50	50	29.72	24.2	18.23
37	Asian Male	68	169.95	50	50	27.45	27.22	19.88
38	Asian Female	68	156.94	50	50	28.48	23.19	17.47
39	Caucasian Male	68	178.1	75	50	28.58	29.58	21.07
40	Caucasian Female	68	163.3	75	50	30.54	24.29	18.21
41	African Male	68	179.9	75	50	28.82	29.92	21.31
42	African Female	68	164.81	75	50	30.65	24.37	18.27
43	Asian Male	68	169.95	75	50	27.28	28.02	20
44	Asian Female	68	156.94	75	50	29.41	23.36	17.51
45	Caucasian Male	68	178.1	75	25	28.63	30.03	20.96
46	Caucasian Female	68	163.3	75	25	31.42	23.77	18.18
47	African Male	68	179.9	75	25	28.86	30.37	21.2
48	African Female	68	164.81	75	25	31.53	23.85	18.25
49	Asian Male	68	169.95	75	25	27.38	28.48	19.89
50	Asian Female	68	156.94	75	25	30.29	22.84	17.49
51	Caucasian Male	68	178.1	25	75	28.83	28.53	20.95
52	Caucasian Female	68	163.3	25	75	29.52	24.19	18.17
53	African Male	68	179.9	25	75	29.09	28.87	21.19
54	African Female	68	164.81	25	75	29.62	24.28	18.23
55	Asian Male	68	169.95	25	75	27.48	26.96	19.88
56	Asian Female	68	156.94	25	75	28.38	23.26	17.47
57	Caucasian Male	68	178.1	50	75	28.8	28.76	21.11
58	Caucasian Female	68	163.3	50	75	29.09	24.79	18.21
59	African Male	68	179.9	50	75	29.06	29.1	21.34
60	African Female	68	164.81	50	75	29.2	24.88	18.27
61	Asian Male	68	169.95	50	75	27.46	27.19	20.04
62	Asian Female	68	156.94	50	75	27.96	23.86	17.51
63	Caucasian Male	48	180.49	50	50	28.92	28.95	21.16
64	Caucasian Female	48	165.51	50	50	29.77	24.35	18.3
65	African Male	48	182.27	50	50	29.18	29.3	21.39
66	African Female	48	167.01	50	50	29.87	24.43	18.36
67	Asian Male	48	172.33	50	50	27.57	27.38	20.09
68	Asian Female	48	159.14	50	50	28.63	23.42	17.6
69	Caucasian Male	48	180.49	75	50	28.68	29.77	21.29
70	Caucasian Female	48	165.51	75	50	30.77	24.64	18.34
71	African Male	48	182.27	75	50	28.92	30.11	21.53
72	African Female	48	167.01	75	50	30.75	24.72	18.41
73	Asian Male	48	172.33	75	50	27.39	28.2	20.22
74	Asian Female	48	159.14	75	50	29.51	23.71	17.65
75	Caucasian Male	48	180.49	75	25	28.74	30.3	21.17
76	Caucasian Female	48	165.51	75	25	28.57	23.97	18.32
77	African Male	48	182.27	75	25	28.97	30.64	21.41
78	African Female	48	167.01	75	25	31.75	24.05	18.38
79	Asian Male	48	172.33	75	25	27.48	28.74	20.1
80	Asian Female	48	159.14	75	25	30.51	23.04	17.62
81	Caucasian Male	48	180.49	25	75	28.96	28.8	21.16
82	Caucasian Female	48	165.51	25	75	29.74	24.39	18.3
83	African Male	48	182.27	25	75	29.23	29.14	21.4
84	African Female	48	167.01	25	75	29.84	24.48	18.36
85	Asian Male	48	172.33	25	75	27.62	27.22	20.1
86	Asian Female	48	159.14	25	75	28.6	23.46	17.6
87	Caucasian Male	48	180.49	50	75	28.82	28.99	21.31
88	Caucasian Female	48	165.51	50	75	29.48	24.98	18.34
89	African Male	48	182.27	50	75	29.08	29.33	21.54
90	African Female	48	167.01	50	75	29.59	25.06	18.4
91	Asian Male	48	172.33	50	75	27.49	27.42	20.24
92	Asian Female	48	159.14	50	75	28.34	24.05	17.64

### Adding artificial limb motion

For the assessment of motor functioning, it is necessary to have available dynamic samples including limb motion. Thus, we are generating four specific right UL movements to add dynamics to the static synthetic human models created in Blender. For that purpose, every synthetic patient produced by the MB-Lab add-on integrates standard skeletons with a well tested rigging, developed to match most external applications and motion capture files.

**Figure 4:**
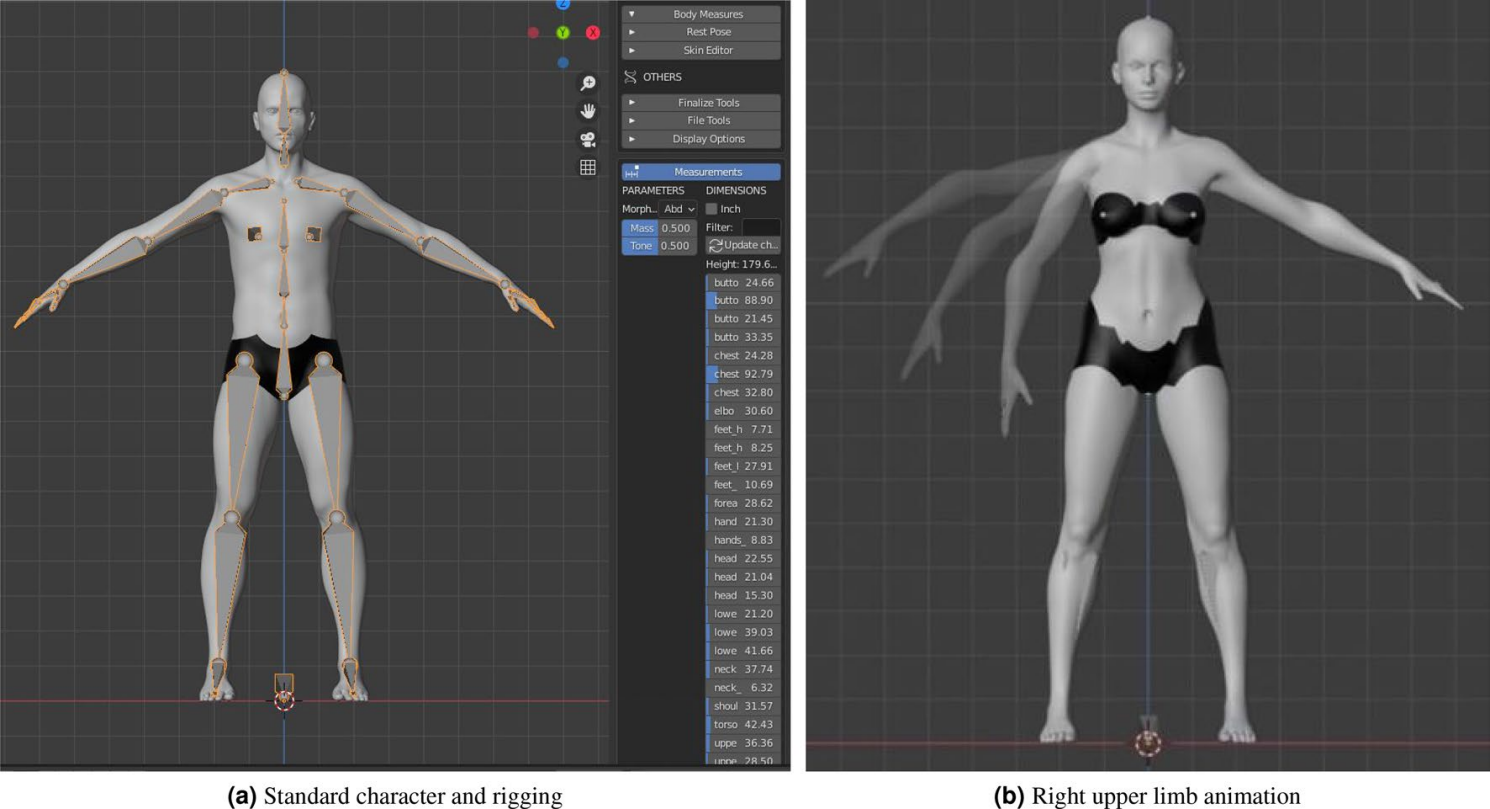
Description of rigging and body animation in blender.

In Fig. 4, a representation of the patient rigging and the menu for modifying the body measure are shown. The skeleton model includes 71 joints, which can be divided into 3 main subgroups: 6 markers for each lower limb, 24 markers for each upper limb and 11 markers for body core, head and neck. The names of the markers are predefined, but they can be changed if the study requires a different labelling. Furthermore, the skeleton model integrates the inverse kinematics being ready for animation.

For animating the synthetic models, it is necessary to define some movement characteristics such as the motion speed and the range of motion. Regarding the movement speed, previous studies have explored the duration for rehabilitation movements and determined about 2–3 s in each movement^37,38^. Considering all of this evidence, we limited the complete motion sequence to 6 s in total since we have two movements involved. Note that a complete motion sequence is understood as the round trip motion. Therefore, a static starting position is defined and recorded as initial pose. The time scroll is placed at three seconds and manually, we move the right UL to the full range of motion and record this medium static pose. Following, the time scroll will be set to six seconds and the model will be moved again to the initial static pose. After recording these points, Blender will automatically animate the model developing a dynamic movement reaching each one of the three recorded static positions.

**Table 2:** Specific UL movements considered in this study.

Joint	Movement	Illustration
Shoulder	Flexion–extension	
	Abduction–adduction	
Elbow	Flexion–extension	
Forearm	Pronation–supination	

Furthermore, four movements were chosen as the one most commonly used in the performance of activities of daily living. The chosen motions are illustrated in Table 2. There are a number of large cross-sectional studies which suggested these movements in rehabilitation activities^39–44^, and a broadly similar point has also recently been made by this researches^45–52^. All of the studies reviewed here support the selection of this four movements because they are representative for assessment and rehabilitation purposes in Spasticity. However, other movements could be added or removed in order to better adapt them to the study following the same method explained before.

As previously mentioned, the chosen set of movements represent most of the range of motion (ROM) of the UL^53–55^ and we can consider them a representative sample of daily activities. For elbow flexion/extension motion, the arm starting position will be completely extended adjacent to the body thorax, the wrist rotates 90$^{\circ }$ facing the hand palm to the shoulder and a 140$^{\circ }$ ± 20$^{\circ }$ flexion will be performed. For shoulder flexion/extension and adduction/abduction, the arm starting position will be also completely extended adjacent to the body thorax with the particularity that in flexion movement, the wrist rotates $-90^{\circ }$ facing the back of the hand to the shoulder. A 90$^{\circ }$ ± 10$^{\circ }$ motion will be performed in both movements. For forearm pronation/supination, the arm starting position will be completely extended, creating 90$^{\circ }$ between the arm and the body thorax, the wrist rotates $-90^{\circ }$ facing the back of the hand to the shoulder and a forearm rotation of 150$^{\circ }$ ± 10$^{\circ }$ will be performed. In all motions, the arm will return to its starting position to complete the movement.

Overall, the benefit of this approach is that we could use these four movements not only for spasticity modelling, but it also for another shoulder or elbow injuries as Adhesive capsulitis, Rotator Cuff Tear, Lateral Epicondylitis or Carpal tunnel Syndrome among others. Furthermore, the robotic arm rehabilitation system presented in Fig. 1 involves UL tasks where these four movements fit accordingly to the robotic arm range performance.

### Dataset result

The obtained dataset may be divided into four main sub-groups. The first sub-group includes the Blender models without animation includes the patients from Blender with MBLab add-on introduced in Table 1 in .blend format. The second sub-group includes the Blender models with animation still in .blend format incorporate the four movements in Table 2 to the 92 patients. Motionbuilder import fbx convert the files in .blend format into .fbx format preparing the data for future transformations.

The third sub-group includes a file format conversion using Motionbuilder for adapting the file format to the motion analysis software. Motionbuilder is employed in our research just because of the special input required in OpenSim. If the final application admits the many export outputs from Blender, this step and the following could be avoided. For OpenSim Trc, we need to first adjust the output from Motionbuilder import fbx to the OpenSim input in .trc. This process may be divided into four main activities.

1. First, the global axis from Blender does not match the global axis from OpenSim. For this reason, we have to rotate on Y axis -90$^{\circ }$.
2. Second, the height of the Y axis in our patient samples is too distant from the Y axis in our OpenSim model. Therefore, a regulation to pair both axis is recommended.
3. Third, the joint names from Blender could not couple the joint names from our OpenSim model and we could need to modify them.
4. Fourth, the export format from Blender is .Fbx and the import format from OpenSim is .Trc. An auxiliary software named MotionBuilder is used to convert from one format to the final one.

**Figure 5:**
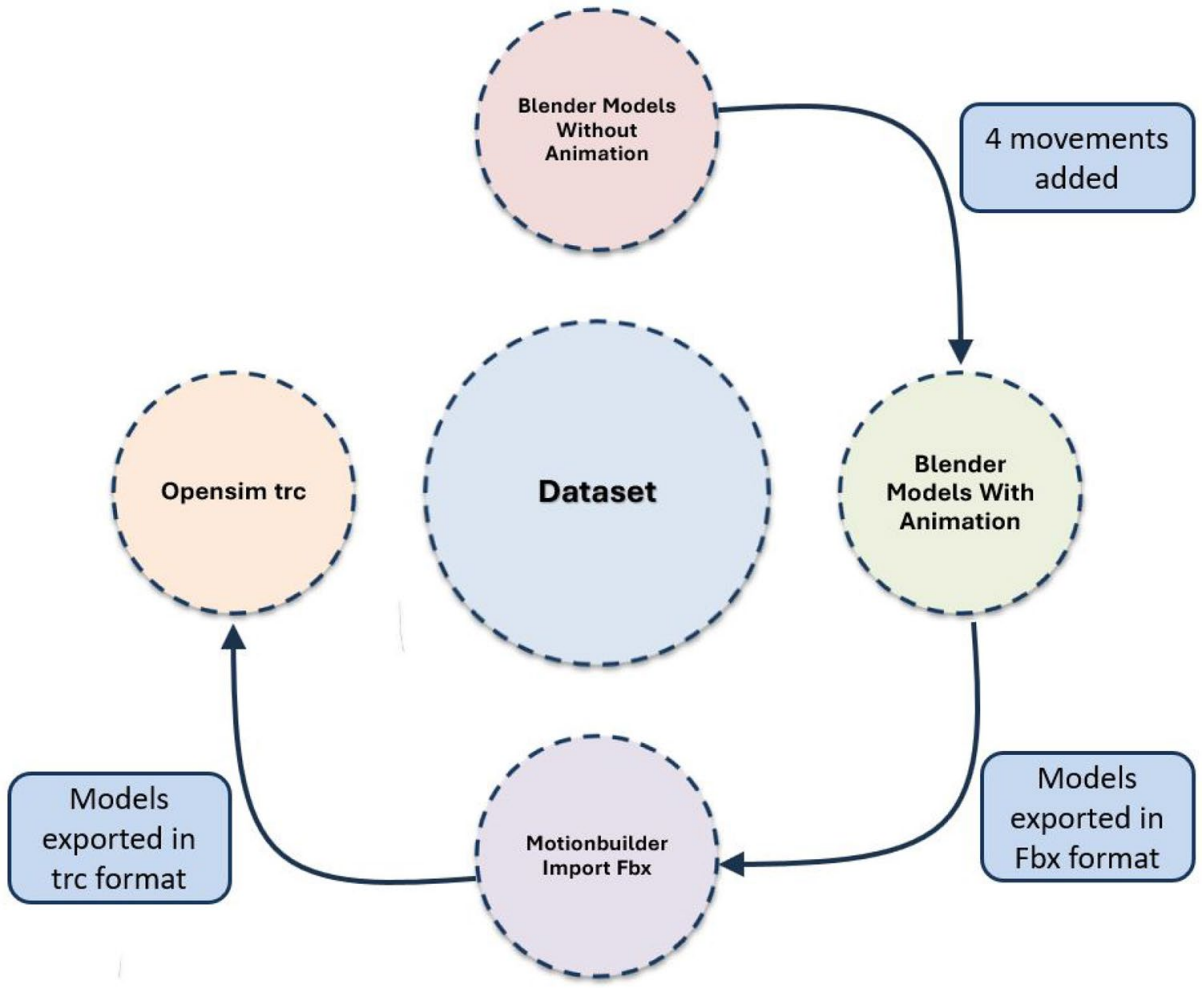
Dataset organization.

Opensim trc folder contains the files already transformed in .trc. The markers generated in Blender for each joint are included also in the dataset structure as a Markers equivalence.txt in the Opensim trc folder to better handle the samples. Blender creates 71 markers for each model and our study only needs 14 markers for our Opensim model. A initial line with the Blender markers is introduced and then, a line with the 14 modified markers to couple the Opensim model joint names. The final number of markers needed will be established by the Opensim model. After finishing all this process, samples are ready to manipulate and the dataset structure is the following in Fig. 5, being this the fourth dataset sub-group.

All these transformations between software formats, axis and markers are necessary to export the Blender synthetic data to OpenSim. If the modelling software supports the OpenSim format or the musculoskeletal software imports the Blender output, we could avoid these laborious steps.

Therefore, 92 static synthetic human body models were created in Blender with MB-Lab add-on and afterwards, four artificial limb motions were selected as representative and added to each human body model generating 368 motions in total. If this quantity of human body models or motions is not acceptable for the investigation, we could add or modify them to ensure adequate results. All samples will be public with free access for the use in future research. The patient dataset and the movements could be modified subject to the demands of the research.

The complete dataset can be found at the following public repository as a contribution for future research: https://doi.org/10.21950/CWZNVC.

## Proposed framework for spasticity modelling

**Figure 6:**
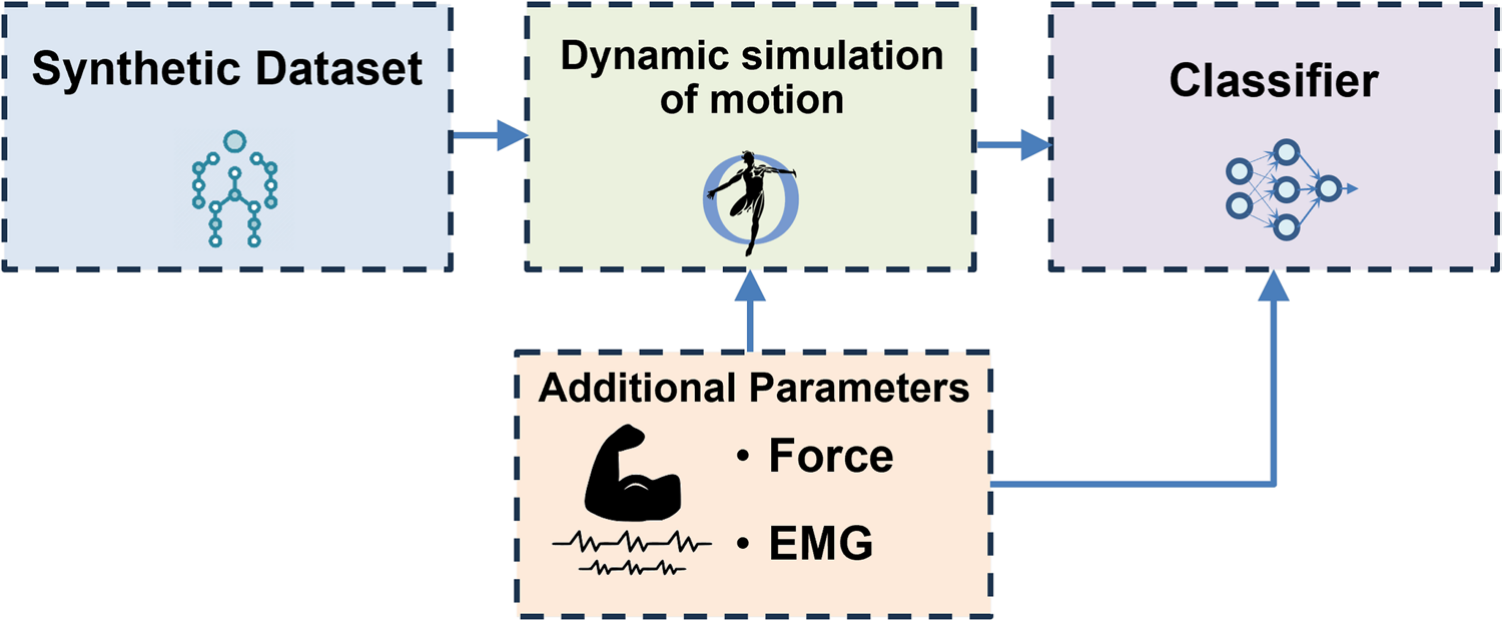
Framework for spasticity measurement.

Once the synthetic data were generated, the final stage of our study comprised a musculoskeletal analysis and a spasticity classifier to apply the generic dataset into our specific Spasticity application. Also, a Force addition and a movement constraint adjustment will be incorporate in our analysis to ease the work for future studies although this is out of the scope of our research. This 2 subsections are natural evolution of our work but in this study are mainly illustrative. Figure 6 illustrates the proposed framework for UL spasticity modelling by using the synthetic dataset.

### Dynamic simulation of spastic motion

To begin this process, OpenSim software was employed based on its reliability and validity in Musculoskeletal Analysis^56,57^. Not only OpenSim’s state of maturity has been proven during all these years but it also the simulation potential and the modularity. There is a widespread community improving the software and several tested models that can be profitable. These advantages made OpenSim the software of choice over other recent musculoskeletal software. Although, Spasticity simulation in each one of the 368 movements is demanded to obtain a Spasticity model. During the past 40 years, much more information has become available on the scales of Spasticity^6,58^. Overall, there seems to be some evidence to indicate that the Modified Ashworth Scale (MAS) is the most extended^59,60^. For this reason, we are building a special plugin for OpenSim based on the plugin already developed by^61^ with the 6 levels of Spasticity from the MAS. Table 3 presents the MAS scoring used in this study.

**Table 3:** Modified Ashworth Scale grades for spasticity.

Grade	Description
0	No increase in muscle tone
1	Slight increase in muscle tone, with a catch and release or minimal resistance at the end of the range of motion when an affected part(s) is moved in flexion or extension
1+	Slight increase in muscle tone, manifested as a catch, followed by minimal resistance through the remainder (less than half) of the range of motion
2	A marked increase in muscle tone throughout most of the range of motion, but affected part(s) are still easily moved
3	Considerable increase in muscle tone, passive movement difficult
4	Affected part(s) rigid in flexion or extension

It is important to highlight that different movements involve different muscle activation. OpenSim allows to personalise and select the muscles involved in a specific movement, according to the constrains of the study. For the four motions chosen, the muscle affected are listed in Table 4.

**Table 4:** Muscles involved in UL motion.

Motion	Muscle
Elbow flexion	Brachial biceps (BICLong, BICShort) and anterior brachialis (BRA).
Pronation	Pronator Teres (PT) and pronator quadratus (PQ).
Supination	Brachial biceps (BICLong, BICShort), brachioradialis and supinator (SUP).
Shoulder, transverse axis—flexion	Anterior deltoid fibers (DELT1), biceps (BICLong, BICShort), pectoralis major (PECM1, PECM2, PECM3).
Shoulder, transverse axis—extension	Rear Deltoid Fibers (DELT3), and Triceps Long Head (TRILong).
Shoulder, sagittal axis—adduction	Teres Major (TMAJ), Pectoralis Major (PECM1, PECM2, PECM3), and Triceps Long Head (TRILong).
Shoulder, sagittal axis—abduction	Deltoid middle fibers (DELT2), supraspinatus (SUPSP) (very important because it initiates it).

The plugin was modified with Visual Studio and adds 3 new characteristics to the muscle: gain factor, threshold value, and time delay that can be added to any muscle in an .osim model. The spastic muscle will show spastic behavior in OpenSim. Our generic muscle model in OpenSim has been gathered from SimTk.org and includes all the right upper limb muscles and other essential muscles for the movement as PECM1, PECM2, PECM3 or DELT1, DELT2, DELT3 (see Table 4). However, only the muscles involved in our study were employed due to high computing issues with the generic muscle model with all the muscles. The Upper Extremity Dynamic Model incorporates the Millard2012EquilibriumMuscle^62^ that evolved the Thelen2003Muscle^63^ and supported the Spasticity plugin. Consequently, each one of the 92 synthetic patients performing four specific movements will have applied 6 different grades of Spasticity. Therefore, 2.208 samples will be analysed in OpenSim. The procedure will continue with five separable basic OpenSim functions. Firstly, Escalating the generic model to the specific patient. Secondly, performing the Inverse Kinematics (IK) functionality to reproduce the motion in OpenSim. Thirdly, executing the Reduced Residual (RRA) to minimize data errors. Fourthly, the Computed Muscle Control (CMC) operation and lastly, completing the Forward Dynamics (FD) step to analysed the muscle activation.

**Figure 7:**
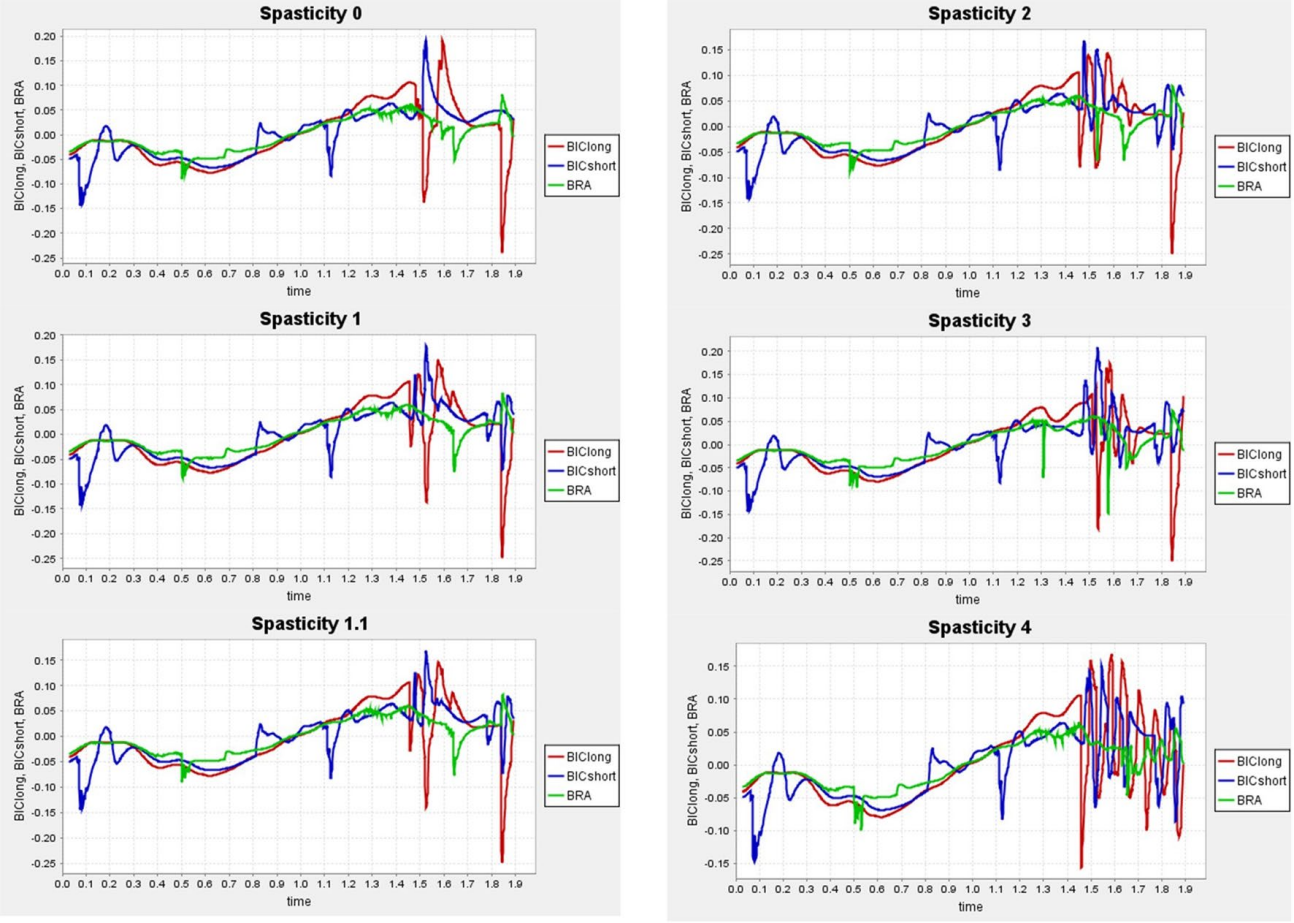
Normalized velocity fiber with MAS spasticity grades for muscles BRA, BICShort and BICLong.

It is important that the markers from the synthetic data match the name of the markers from the generic model to perform a proper scaling. A scaling will be completed to adapt the length of the bones from the generic model to the patient’s and after that, the IK tool with the same scaling .trc file to generate the motion file .mot. The purpose of the RRA is to minimize the effects of modeling and marker data processing errors to avoid any inaccuracy in the next CMC analysis. With the output from the RRA, we will compute the muscle excitations with no external loads nor additional forces applied. The final stage of the process comprehended the FD and obtaining the required files for the neural network modelling. This 2 archive contains the time histories of the model controls and the model states, including joint angles, joint speeds, muscle activations, muscle fiber lengths, etc. We will work with them to create the spasticity model. Figure 7 represents the 6 levels of Spasticity described in the MAS scale in Table 3. An elbow flexion movement with the first Caucasian male patient with 50% mass and 50% tone was performed. Among the multiple outputs from the musculoskeletal analysis, the normalized velocity fiber in BRA, BICShort and BICLong muscles was selected to illustrate the variation. From the above Fig. 7, we can see how mostly at the end of the movement, muscle activation differs between the diverse Spasticity grades.

### Musculoskeletal analysis improvement

One of the features of OpenSim when developing models of musculoskeletal structures and create dynamic simulations of movement is that external forces can be included in the analysis. This section aims to illustrate the possibilities of adding forces from human-robot interaction and other movement constraints to adjust the range of motion according to the patient needs. Note that this section only highlights the potential of OpenSim to improve the neuromusculoskeletal system simulation using real data.

#### Force addition

In our case, we collected human-robot interaction forces from our Roboticslab Arm rehabilitation solution equipped with a handler located on the tool centre point (TCP) of the robotic arm. A collaborative lightweight robot IIWA from KUKA is employed for this purpose due to the high-precision joint torque sensors. Additionally, a force-torque (F/T) sensor is included at the robot TCP. According to the setup presented in Fig. 1, we performed two phases alternating the clinician and patient roles. In the first step, the robot will be in no-gravity state and with no external forces. The clinician will guide the robot and we will collect the data from the movement and the data from the forces of the robot. In the second step, the patient will grab the handler and the robot will replicate the first movement recording the force opposition in the handler. The IIWA robot was chosen to record the forces but alternate methods as inertial measurement units (IMU) with Electromiography electrodes (EMG), postural control through kinect sensor or other force evaluation system can be used. Once we have the results, the forces applied from the IIWA in the first movement are subtracted to the second movement. Consequently, only the forces from the patient will be included in OpenSim. A major advantage of this concept is that can be duplicated for any movement that the clinician considers it appropriate.

**Figure 8:**
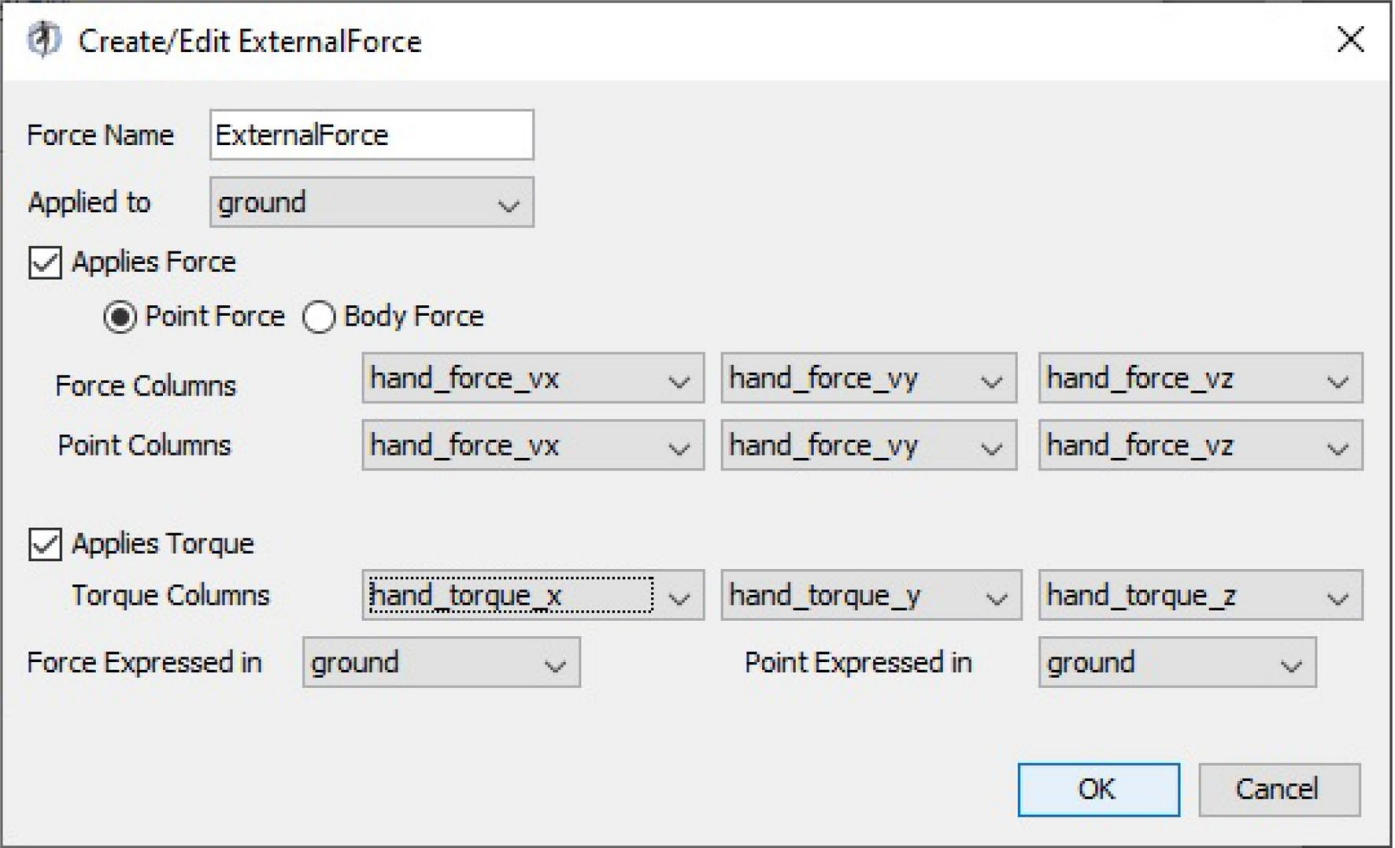
External forces addition in OpenSim.

To transfer the data gathered to OpenSim, the Robotics System Toolbox add-on from MATLAB will be the software used to transform the data into force vectors and torques. We will storage the data in a .mot file for the future use in the CMC tool in OpenSim. The CMC tool will consider this external loads automatically in the computing. We will apply the external forces to the marker “WristRight” because this marker is the most related to the physical position of the handler. Following the procedure in the manual, we will create and add an external force in OpenSim software as shown in Fig. 8.

#### Movement constraints

Another important characteristic of OpenSim is that movement constraints could be modified. We are not modifying any constraints in this research, but it could be beneficial for future research when the movements are not in the model’s limit. For adjusting the model constraints, first we need to comprehend why and where are the body physical limits to transfer this consciousness to the OpenSim simulation.

**Figure 9:**
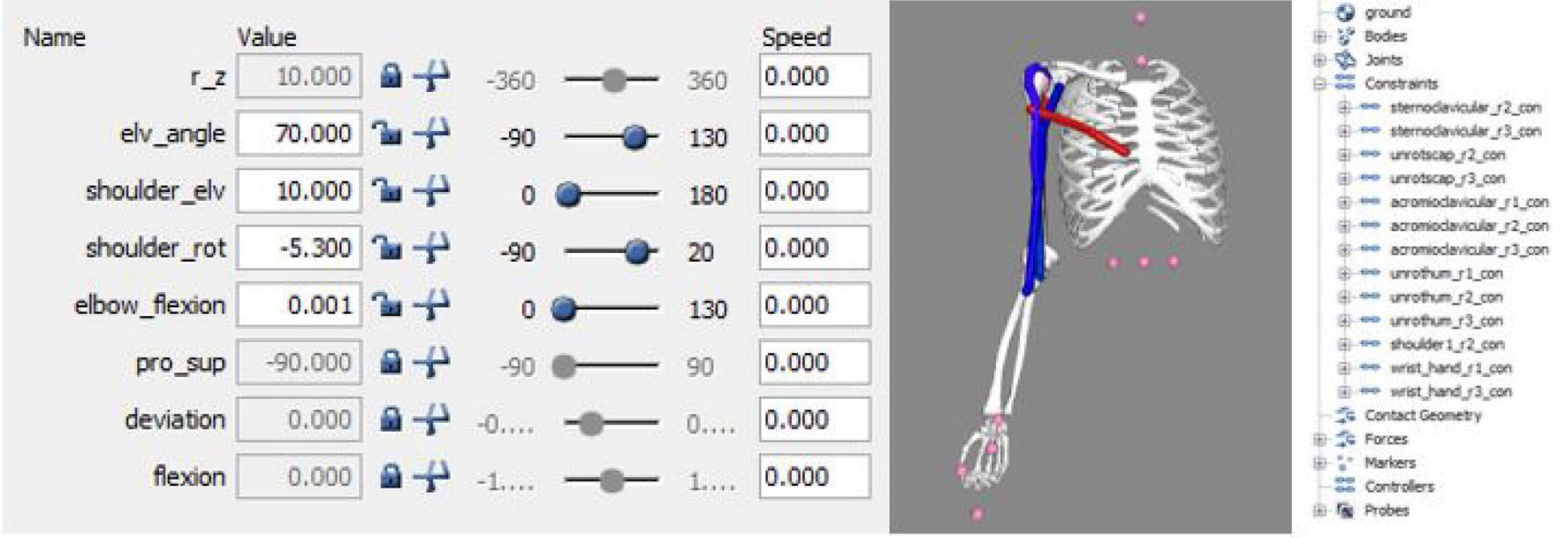
Model constraints.

Upon that, we will open the .osim model file with a text editor and locate the ConstraintSet area. As we can see in Fig. 9, the bones and joints are connected between them to simulate the human body and we will respect this limits. Constraints angle are expressed in radians.

## Discussion

Currently, the assessment of motor functionality is performed by clinicians using standardised tests towards objective evaluation. Since the process is manually performed, the evaluation may include some degree of uncertainty (subjectivity) that may come from movement variability, appreciation (inter-operator), etc.^64^. Most evaluation tests are composed of well-defined exercises (or procedures) based on numerical scales, which may be susceptible of automation^65^. An example is the assessment of spasticity, which is strongly linked to the clinician’s expertise (thereby, it is affected by inter-operator variability) and gives the results in terms of a numerical scale, the MAS.

In this context, robot-aided systems have been proposed as an alternative method to manual procedures of spasticity quantification^6^. Robot-assisted strategies provides some advantages such as reliable data collecting of patient’s performance, accurate limb mobilization, high repeatability in trajectories, etc. However, the high complexity of this technology reduce the possibilities of deployment in healthcare facilities and, therefore, the options to collect data from real patients, which is essential to classify and understand the behaviour of motor control problems. This fact motivates this paper to search for an alternative method to solve the lack of real patient samples.

Traditionally, the most common procedure for acquiring movements from a real patient was the Motion Capture (MOCAP). MOCAP systems can capture subtle nuances and details of human motion, and create realistic and natural animations. However, the main limitation of traditional MOCAP is the number and variety of samples. MOCAP samples are limited to real patients willing to collaborate with the investigation and does not include all types of Spasticity or human body characteristics. By contrast, our proposed 3D synthetic generation can produce as many samples as the system requires with unique and different attributes. Moreover, the actual subjectivity in manual evaluations could lead to undesired mistakes and our proposed system could minimize inter-operator variability because the clinician evaluation will be supported by objective data and a standard assessment process to avoid inaccuracies. Compared with traditional methods where all types of patients were required in order to build a robust model, these results indicate that an accurate model of Spasticity can be developed with an alternate and modern method as 3D modelling.

The use of synthetic data and machine learning models in clinical settings decreases the ethical concerns not only in patient confidentiality and anonimity, but it also of the potential for harm for each volunteer^66^. Furthermore, patient comfort and safety during the evaluation process will increase due to the precision sensors in the robot-aided systems. A constant measure of forces can be designed to prevent any harmful movement of the patient. This standard assessment could be particularly useful for tracking the long-term progression of degenerative diseases^67^. Small alterations between evaluations combined with the big data and results digitization could improve the tracking. Also, the proposed system is based on existing scale evaluation. Therefore, the integration of the robot-aided system into the existing clinical workflow might not constitute an issue. However, a short training is necessary for healthcare providers in order to fully understand the complete robot-aided system.

Despite human synthetic data can be easily generated to replace human patients, a major source of uncertainty is in the Spasticity simulation and how differs from the real patient situation. Using synthetic data, the neural network produced a most adequate output when a heterogeneous and higher quantity of data input is given. Although these results are not crossed with real patients, a natural progression of this work will be validate them with real data in future studies. The Spasticity model is not limited to the upper limb and could be exported to lower limb. Consequently, the motion production is also not limited to 4 movements and it can be modified, deleting or adding new ones, due to the necessity of the research. MAS is addressed in the investigation based on the current clinical procedure and the reliability of the scale during the years although additional research is needed to better understand the Spasticity nature. A short introduction for adding forces and modify constraints has been included in order to complete and improve the addition of real patients to the study.

Another relevant line to explore is the integration of performance-based data in the Spasticity simulation. A proposed method of measuring real patient performance would be recording the movement with a Kinect camera, a Motion Capture system if available, or placing EMG’s in the arm joints to estimate the normalized fiber velocity^68,69^. The articular coordinates of each joint would be collected and a proper scaled and inverse kinematics in OpenSim could be accomplished. Moreover, the EMG sensors could register the muscle behaviour and the developed spasticity plugin would be adjusted to achieve better results.

The current study is limited by the absence of formal data validation and further research should focus on adding real patients to support the good results of our investigation. In contrast to simulation, real trajectories from human patients may introduce variations and flaws into the movements. Also, an additional uncontrolled factor is the muscle compensation during the motion and more information on its behaviour would help us to establish a greater degree of accuracy on the results. These issues must be analysed in future work for validating the model with real patient data to ensure its clinical relevance. Furthermore, the synthetic data generated are only taking into consideration 3 ages, 4 predefined phenotypes and a specific relation between mass and tone. More synthetic samples could be added to Table 1 expanding the range of age, not limiting only to predefined phenotypes and body measures and enlarging the mass-tone relation. For the musculoskeletal analysis, the type of muscle selected during the simulation and the spasticity plugin limited the final results. A different type of muscle could be incorporated to compare the output. Notwithstanding these limitations, the proposed synthetic data generation method could provide a guide for future dataset development in clinical applications beyond Spasticity.

## Conclusions

The purpose of the current study was to determine the viability of synthetic data generation for clinical purposes. The results of this research support the idea that 3D anatomical recreation could be effective in the field of modelling musculoskeletal injuries. Prior to this study, it was difficult to make predictions about how possible were to export human body samples to manipulate in OpenSim, but the results lays the groundwork for future combination of VR and sensorimotor impairment modelling. Once again, collaboration between researchers and medical practitioners is critical in order to build appropriate systems that support the development of new and profitable methods in the clinical area.

Further research needs to be done to establish how Spasticity simulation changes the model because one source of weakness in this study which could have affected the measurements was the quality of Spasticity plugin and the absence of real samples. A reasonable approach to tackle this issue could be to validate the model with selected real patients because more information on real cases would help us to establish a greater degree of accuracy on this matter. Therefore, the challenge now is to develop a robust and accurate plugin verified with real patients that allows modelling in OpenSim not only for Spasticity but it also for other motor disorders as Parkinson’s. The empirical findings in this study made available for all researches provide a basis for future studies.

Also, a natural progression of this work will be analyse the possibility to generate the synthetic data with an efficient and faster method. The complete process of generation, transformation and computation requires a large amount of time and consumes a lot of computational resources, becoming one of the main areas for improvement. OpenSim provides the opportunity to create new models with python^70^ and we could accomplish specific models with predefined movements with no need of an additional software and a fully automate workflow.

## Data Availability

The whole dataset generated in this work is available at: https://doi.org/10.21950/CWZNVC.
